# A lesion extending three or more slices as a predictor of progressive infarction in anterior circulation small subcortical infarction

**DOI:** 10.3389/fneur.2022.926187

**Published:** 2022-10-05

**Authors:** Jing Lin, Xiaocheng Mao, Yunfang Liao, Si Luo, Qin Huang, Ziwei Song, Shumeng Li, Chengjin Li, Yuexin Qiu, Yuhang Wu, Min Zhu, Xiaobing Li, Qiulong Yu, Daojun Hong

**Affiliations:** ^1^Department of Neurology, The First Affiliated Hospital of Nanchang University, Nanchang, China; ^2^Department of Pharmacy, The First Affiliated Hospital of Nanchang University, Nanchang, China; ^3^Jiangxi Province Key Laboratory of Preventive Medicine, School of Public Health, Nanchang University, Nanchang, China

**Keywords:** progressive infarction, early neurological deterioration (END), subcortical infarction, infarct diameter, infarct slice

## Abstract

Progressive infarction (PI) is common in small subcortical infarction and may lead to a poor outcome. The purpose of our study is to identify neuroimaging predictors for PI. From April 2017 to December 2020, we enrolled 86 patients with an anterior circulation subcortical infarction within 48 h after onset. Progressive infarction was defined by an increase of ≥ one point in motor power or ≥ two points in the total National Institute of Health Stroke Scale score within 7 days after admission and further confirmed by diffusion-weighted imaging (DWI). To identify predictors, demographic characteristics, clinical information, laboratory date, and neuroimaging characteristics were evaluated. The infarct size and infarct slice number were measured by DWI. We found that thirty-one patients (36%) had PI. In a univariate analysis, the patients with PI had higher levels of triglyceride, lower levels of blood urea nitrogen and prothrombin time, and a higher frequency of infarct slice number ≥ three compared to the patients without PI. After logistic regression stepwise adjustment for all considered relevant confounders, infarct slice number ≥ three slices proved to be independently associated with PI (OR = 4.781, 95% CI 1.677–13.627; OR = 4.867, 95% CI 1.6–14.864; OR = 3.584, 95% CI 1.034–12.420). Our study showed that a lesion extending ≥ three slices on DWI is an independent predictor for progressive infarction in patients with anterior circulation small subcortical infarction.

## Introduction

Early neurological deterioration (END) occurs in up to one-third of patients with acute ischemic stroke after hospital admission and is associated with worse outcomes (1). A variety of pathophysiological mechanisms cause END, including progressive infarction (PI), increased intracranial pressure, recurrent cerebral ischemia, and secondary parenchymal bleeding (2). Among them, progressive infarction is the most frequent cause, accounting for ~30% (2).

Small subcortical infarction (SSI), regarded as the small vessel occlusion subtype in stroke classification, usually has a small infarction volume and leads to relatively limited functional deficits (3, 4). However, between 20 and 30% of SSI patients experience END, which severely affects their functional prognosis (5, 6). According to the pathophysiological mechanism, Fisher and Caplan divided SSI into two main types: lacunar infarction (LI) and branch atheromatous disease (BAD) (7, 8). In recent years, several studies have reported that patients with BAD are more likely to develop worsening neurological deficits than patients with LI (9–11). Distinguishing between these two entities is of clinical importance, but knowledge of this distinction is limited. Branch atheromatous disease is usually defined as a lesion extending three or more consecutive slices or an infarct diameter ≥15 mm in diffusion-weighted imaging (DWI). Lacunar infarction is usually considered as a single lesion of <15 mm in the greatest diameter within the territory of the brainstem or basal penetrating arteries (9, 10). However, with the development of neuroimaging, inconsistencies have been found in the radiological differentiation between BAD and LI by the diameter and slice number of the infarction (12, 13). In addition, from the clinical perspective, brainstem infarction belongs to the small vessel occlusion subtype of stroke that rarely extends three slices.

In the present study, we did not attempt to distinguish between BAD and LI to investigate and purely focus on the relationship between the neuroimaging characteristics of anterior circulation small subcortical infarction and progressive infarction.

## Subjects and methods

### Patients

Between April 2017 and December 2020, we selected patients from a registry of patients consecutively admitted to the Stroke Unit of the First Affiliated Hospital of Nanchang University. All procedures were approved by the Ethics Committee of the First Affiliated Hospital of Nanchang University. Patients were recruited if they met the following criteria: (1) were admitted to our hospital within 48 h after symptom onset and (2) had evidence of an anterior circulation subcortical infarction on DWI that was consistent with the clinical deficit (anterior circulation subcortical infarction was defined as the infarct supplied from the lenticulostriate artery from the middle cerebral artery or the Heubner's artery from the anterior cerebral artery). The exclusion criteria were as follows: (1) patients with cortical infarcts or multiple subcortical infarcts; (2) patients with infarcts of posterior circulation; (3) patients with evidence of other etiologies leading to ischemic strokes, such as vasculitis, dissection, cardioembolism, moyamoya disease, and patent foramen ovale; and (4) patients with imaging data that could not be analyzed, a lack of laboratory data or a lack of follow-up. Early neurological deterioration was defined as an increase of ≥1 point in motor power or ≥2 points in the total National Institute of Health Stroke Scale score within 7 days after admission (14). More important, the progression of infarction needed to be confirmed by DWI.

### Definition of clinical information

Data were collected on demographic characteristics and clinical information that included age, sex, history of hypertension and diabetes, initial National Institutes of Health Stroke Scale (NIHSS) score, discharge NIHSS score, and modified Rankin scale (mRS) score at 90 days. The mRS was evaluated by in-person interviews or telephone. The laboratory data were evaluated within 24 h of admission, including the neutrophil count, lymphocyte count, fasting blood glucose level, total cholesterol level, triglyceride level, high-density lipoprotein cholesterol level, low-density lipoprotein cholesterol level, homocysteine level, blood urea nitrogen (BUN) level, creatinine level, prothrombin time (PT), prothrombin time ratio, prothrombin time activity, international standardized ratio, activated partial thromboplastin time, fibrinogen level, thrombin time, D-dimer level, and urine specific gravity.

### Evaluation of neuroimaging information

All enrolled patients underwent MRI on a 3.0 Tesla scanner (MAGNETOM Trio, Siemens, Erlangen, Germany) within 48 h of onset and were immediately reevaluated once neurological deterioration was detected. The protocol included DWI (TR/TE of 3,100/91 ms; field-of-view 230 × 230 mm^2^; 19 slices with slice thickness of 5 mm; voxel size = 1.2 × 1.2 × 5 mm^3^; 2b values of 0 and 1,000 s/mm^2^; scan time of 1.16 min), T1-weighted imaging (TR/TE of 250/2.46 ms; Field of view 220 × 220 mm^2^; 19 slices with slice thickness of 5 mm; voxel size = 0.9 × 0.7 × 5 mm^3^; 2b values of 0 and 1,000 s/mm^2^; scan time of 1.12 min), T2-weighted imaging (TR/TE of 4,000/113 ms; Field of view 220 × 220 mm^2^; 19 slices with slice thickness of 5 mm; voxel size = 0.7 × 0.7 × 5 mm^3^; 2b values of 0 and 1,000 s/mm^2^; scan time of 1.14 min), fluid attenuated inversion recovery (FLAIR) (TR/TE of 8,000/79 ms; Field of view 220 × 220 mm^2^; 19 slices with slice thickness of 5 mm; voxel size = 1.1 × 0.9 × 5 mm^3^; 2b values of 0 and 1,000 s/mm^2^; scan time of 1.38 min), and three-dimensional time-of-flight MRA (TR/TE of 22/3.86 ms; Field of view 235 × 235 mm^2^; voxel size = 0.9 × 0.6 × 0.6 mm^3^; 2b values of 0 and 1,000 s/mm^2^; scan time of 3.12 min). All the images were reviewed and evaluated by two trained neurologists who were blinded to the patients' information. Infarct size was defined as the maximal diameter of the lesion on axial DWI. The infarct slice number was measured as the number of visible infarcts on axial DWI. Leukoaraiosis was analyzed by a 4-point score as proposed by Fazekas et al. (15).

### Statistical analysis

In univariate analysis, we compared differences between two groups using Student's *t*-test or the Mann–Whitney *U*-test for continuous variables and the *chi-square* test or Fisher's exact test for categorical variables. Multivariable logistic regression models were performed to identify possible contributing factors for progressive infarction, including all variables with *P* < 0.05 on univariate analysis and some variables reported to be associated with progressive infarction. Significance levels were set at *P* < 0.05 for 2-tailed tests. All statistical analyses were performed using SPSS version 26.0 (SPSS Inc., Chicago, IL, USA).

## Results

A total of 86 patients (60 men; mean age, 62.9 y) were recruited for analysis. A total of 31 (36%) patients and 55 (64%) patients were classified as PI and non-progressive infarction (non-PI) patients, respectively.

The baseline demographic, clinical, and laboratory characteristics of the two groups are listed in Table 1. Compared with patients without PI, patients with PI had higher NIHSS scores at discharge, higher mRS scores at 3 months after onset, higher levels of triglycerides, and lower levels of BUN and PT (all *p* < 0.05). However, there were no significant differences between these two groups in age, sex, the prevalence of hypertension and diabetes, the neutrophil–lymphocyte ratio, fasting glucose levels, other blood lipid levels, other coagulation function indicators, or urine-specific gravity.

**Table 1:** Comparison of baseline characteristics between PI and non-PI groups.

**Variable**	**PI**	**Non-PI**	***P***
	**(*n* = 31)**	**(*n* = 55)**	
**Demographic characteristics**			
Age, mean ± SD, years	61.23 ± 12.36	63.98 ± 9.91	0.261
Male, *n* (%)	22 (71.0%)	38 (69.1%)	0.856
**Clinical data**			
Hypertension, *n* (%)	19 (61.3%)	30 (54.5%)	0.652
Diabetes, *n* (%)	8 (25.8%)	8 (14.5%)	0.144
Initial NIHSS, median (IQR)	2 (2, 3)	2.0 (1, 4)	0.816
Discharge NIHSS, median (IQR)	3 (1, 4)	1.0 (0, 2)	0.001*
**3-mo mRS**, ***n*** **(%)**			0.031*
0	8 (25.8%)	29 (52.7%)	
1	10 (32.3%)	17 (30.9%)	
2	2 (6.5%)	4 (7.3%)	
3	4 (12.9%)	2 (3.6%)	
4	6 (19.4%)	3 (5.5%)	
5	0 (0%)	0 (0%)	
6	1 (3.2%)	0 (0%)	
**Laboratory data**			
NLR, median (IQR)	2.31 (1.93, 3.36)	2.49 (1.95, 3.46)	0.480
Fasting glucose (mmol/L), median (IQR)	5.65 (5.2, 11.21)	5.45 (4.68, 6.85)	0.104
Total cholesterol (mmol/L), median (IQR)	4.82 (4.21, 5.71)	4.38 (3.79, 5.19)	0.064
Triglyceride (mmol/L), median (IQR)	1.69 (1.37, 2.32)	1.32 (0.91, 1.62)	0.008*
HDL-cholesterol (mmol/L), median (IQR)	1.09 (0.96, 1.32)	1.16 (1, 1.37)	0.284
LDL-cholesterol (mmol/L), median (IQR)	2.84 (2.7, 3.42)	2.7 (2.15, 3.65)	0.064
Homocysteine (umol/L), median (IQR)	14.6 (12, 20)	17.55 (12, 20)	0.332
BUN (mmol/L), median (IQR)	4.1 (3.3, 5.2)	4.7 (3.5, 6.7)	0.039*
Cr (umol/L), median (IQR)	64.3 (54.3, 73.1)	67.1 (52.6, 78.4)	0.284
PT (s), median (IQR)	10.8 (10.3, 11.5)	11.3 (10.8, 11.6)	0.041*
PTR, median (IQR)	0.95 ± 0.06	0.98 ± 0.08	0.178
PTA (%), median (IQR)	111.90 (102.55, 128.55)	106.00 (99.85, 115.20)	0.078
INR, median (IQR)	0.94 (0.90, 1.00)	0.97 (0.92, 1.00)	0.199
APTT (s), median (IQR)	26.80 (23.70, 28.10)	27.20 (26.20, 29.90)	0.116
Fibrinogen (g/L), median (IQR)	2.67 (2.25, 3.16)	2.79 (2.43, 3.3)	0.477
Thrombin time (s), median (IQR)	18.10 (17.45, 18.90)	17.80 (17.15, 18.50)	0.338
D-dimer (mg/L), median (IQR)	0.26 (0.17, 0.51)	0.37 (0.22, 1.14)	0.07
Urine specific gravity, median (IQR)	1.02 (1.01, 1.02)	1.02 (1.01, 1.02)	0.694
**Imaging date**			
Diameter of infarcts (mm), mean ± SD	16.81 ± 6.54	16.35 ± 9.29	0.149
Diameter of infarcts≥ 15 mm, *n* (%)	18 (58.1%)	20 (36.4%)	0.071
Extension ≥ three slices, *n* (%)	25 (80.6%)	25 (45.5%)	0.002*
**Leukoaraiosis**, ***n*** **(%)**			0.068
0	7 (22.6%)	3 (5.5%)	
1	12 (38.7%)	26 (47.3%)	
2	4 (12.9%)	15 (27.3%)	
3	8 (25.8%)	11 (20%)	

PI, Progressive Infarction; NIHSS, National Institute of Health Stroke Scale; mRS, Modified Rankin Scale; 3-mo, 3-months. NLR, Neutrophil–Lymphocyte ratio; HDL, High-Density Lipoprotein; LDL, Low-Density Lipoprotein; BUN, Blood Urea Nitrogen; Cr, Creatinine; PT, Prothrombin Time; PTR, Prothrombin Time Ratio; PTA, Prothrombin Time Activity; INR, International Standardized Ratio; APTT, Activated Partial Thromboplastin Time; IQR, Interquartile Range; SD, Standard Deviation. *p < 0.05.

**Figure 1:**
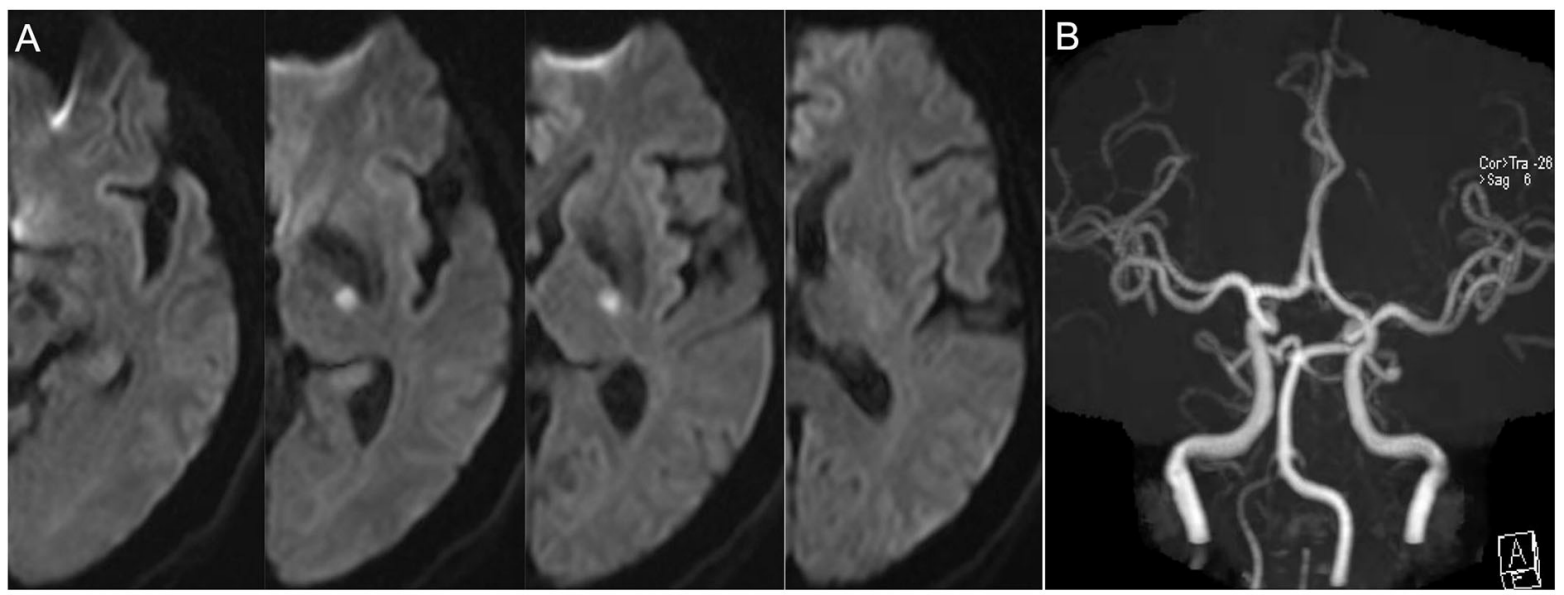
Representative case of non-progressive infarction. **(A)** The infarct slice number <3 on 5 mm thick axial DWI images and the patient had no progressive infarction. **(B)** MRA showed no obvious etiologies (vasculitis, dissection, Moyamoya disease) that led to ischemic stroke.

**Figure 2:**
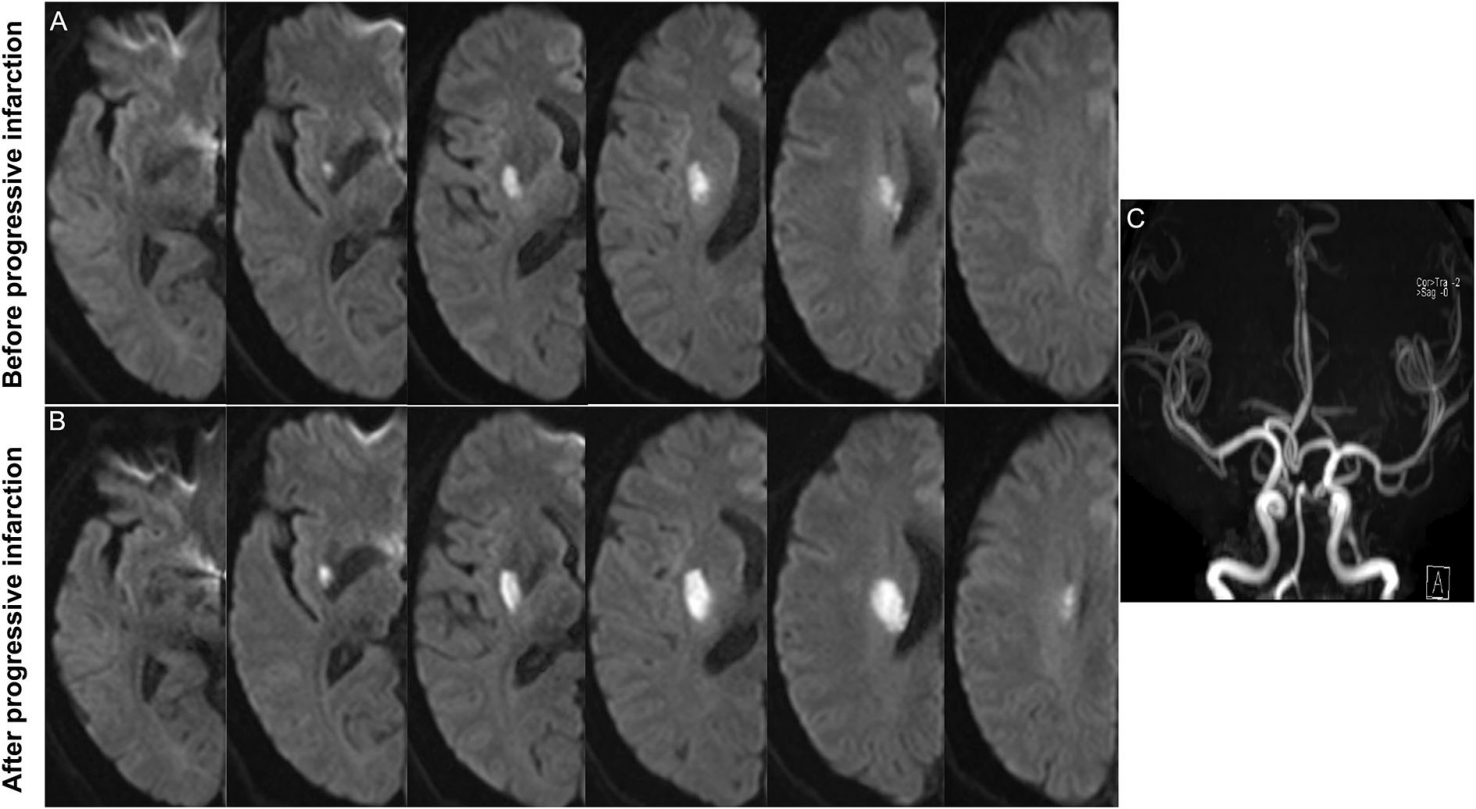
Representative case of progressive infarction. **(A)** Initial axial DWI indicated the infarct slice number was ≥ 3. **(B)** Axial DWI showed the growth of infarction. **(C)** MRA showed no obvious etiologies (vasculitis, dissection, Moyamoya disease) that led to ischemic stroke.

The baseline neuroimaging characteristics, including the infarct diameter, the number of lesion slices, and the leukoaraiosis scores, are also shown in Table 1. There were nearly 1.8 times as many patients with PI with more elongated lesions compared with those without PI (extension ≥ three slices in 80.6 vs. 45.5%, *p* = 0.002) (Figures 1, 2). However, there was no significant difference between the two groups regardless of the infarct diameters or the proportion of infarcts ≥15mm. In addition, the leukoaraiosis scores showed no significant difference between the PI group and the non-PI group.

A multivariable logistic regression model adjusted for age and sex revealed that an extension ≥ three slices was independently related to PI (Model 1: OR = 4.781, 95% CI 1.677–13.627). Furthermore, an extension ≥ three slices was also independently associated with PI after adjustment for triglyceride levels, BUN levels, and PT (Model 2: OR = 4.867, 95% CI 1.6–14.864). At last, after adjusting for relevant confounders, including fasting glucose levels, total cholesterol levels, triglyceride levels, BUN levels, PT, D-dimer levels, an infarct diameter ≥15 mm, and leukoaraiosis scores, an extension ≥ three slices remained significantly associated with PI (Model 3: OR = 3.584, 95% CI 1.034–12.420) (Table 2).

**Table 2:** Associations between infarct extension ≥ three slices and progressive infarction.

	**OR**	**95% CI**	**p**
**Adjusted model** ^**1**^			
Extension ≥ three slices	4.781	1.677–13.627	0.003
**Adjusted model** ^**2**^			
Extension ≥ three slices	4.867	1.6–14.864	0.005
**Adjusted model** ^**3**^			
Extension ≥ three slices	3.584	1.034–12.420	0.044

OR, odds ratio; CI, confidence interval.

^1^Adjusted model was controlled for age, male.

^2^Adjusted model was controlled for triglyceride, blood urea nitrogen, and prothrombin time.

^3^Adjusted model was controlled for fasting glucose, total cholesterol, triglyceride, blood urea nitrogen, prothrombin time, D-dimer, diameter of infarcts >15 mm, and leukoaraiosis.

## Discussion

The main finding of our study was that a lesion extending three or more slices was independently associated with progressive infarction in patients with anterior circulation small subcortical infarction. However, other imaging characteristics, including leukoaraiosis and infarct diameter, were not significantly associated with progressive infarction. In addition, our results demonstrated that patients with progressive infarction had more severe functional disabilities than those without progressive infarction. Our innovative points are as follows: (i) we specifically focused on progressive infarction; not only did the symptoms meet the criteria for early neurological deterioration, but PI was also confirmed by DWI, and (ii) we solely focused on patients with anterior circulation small subcortical infarction.

During the past few years, END has been reported to occur more frequently in BAD-related strokes than in LI. However, the distinction between BAD and LI is mostly based on indirect evidence relying on neuroimaging, although the term BAD was originally created based on pathology (7, 8). Although BAD was considered identifiable by an infarct diameter ≥15 mm and an infarct extension ≥ three slices in most examined work, there is currently no consensus as to the definition of BAD (16). The definitions of BAD used in the three studies focusing on the association between BAD and END were different (9–11). Therefore, we did not distinguish between BAD and LI, but directly investigated the imaging predictors for progressive infarction. At last, we demonstrated that a lesion extending three or more slices was independently associated with PI in anterior circulation small subcortical infarction.

At present, there is controversy about whether an infarction > three slices is a risk factor for END, and several studies have demonstrated different findings. Ohara et al. found that a posterior-type infarct, but not the infarct slice number, was an independent predictor for the progression of motor deficits (17). In a similar manner, Nannoni et al. (18) demonstrated a negative association between a lesion extension ≥ three slices and neurological worsening. Nevertheless, Saji et al. (19) revealed that arterial stiffness, infarct size, and ≥ three infarct slices were all independently associated with progressive neurological deficits. The reasons for this discrepancy may be as follows: (i) the slice thickness on DWI was different; some were 5 mm, and some were 7 mm; (ii) the definitions of END in the related studies were symptomatic definitions, whereas multiple mechanisms are involved in END, including progressive infarction, increased intracranial pressure, recurrent cerebral ischemia and secondary parenchymal bleeding, which may affect the results; and (iii) brainstem infarction rarely exceeds three slices due to vascular anatomy (20), but some studies concurrently recruited anterior and posterior circulation infarction, which may make a difference in conclusions. Our present study defined progressive infarction not only based on symptoms but also, more importantly, on the re-examination of MRI to determine whether END was due to progressive infarction. In addition, we purely focused on anterior circulation and small subcortical infarction and did not include posterior circulation infarction. At last, we found a positive relationship between progressive infarction and ≥ three infarct slices using a slice thickness of 5 mm on DWI.

A limitation of our study was the number of patients in each group. A key point in our enrollment criteria was the definition of progressive infarction, which required not only the aggravation of symptoms but also the confirmation by DWI. Although strict enrollment criteria could accurately determine patients with progressive infarction, they also led to a small sample size.

## Conclusion

In conclusion, a lesion extending ≥ three slices was an independent predictor for progressive infarction in patients with anterior circulation small subcortical infarction. In addition, patients with progressive infarction tend to have worse outcomes. Therefore, it is important to identify the predictors for progressive infarction.

## Data availability statement

The raw data supporting the conclusions of this article will be made available by the authors, without undue reservation.

## Ethics statement

The studies involving human participants were reviewed and approved by Research Ethics Committee of First Affiliated Hospital of Nanchang University. Written informed consent for participation was not required for this study in accordance with the national legislation and the institutional requirements.

## Author contributions

Concept and design: JL, XM, QY, and DH. Acquisition, analysis, or interpretation of data: YL, SLu, QH, ZS, SLi, CL, and XL. Drafting of the manuscript: JL and XM. Critical revision of the manuscript for important intellectual content: QY and DH. Statistical analysis: YQ and YW. Obtained funding: JL and DH. All authors have read and approved the final manuscript.

## Funding

This study was supported by the National Natural Science Foundation of China for Young Scientists (No. 82101405), the National Natural Science Foundation of China (Nos. 81460199 and 82160252), the Natural Science Foundation of Jiangxi Province for Young Scientists (No. 20212BAB216023), the Science and technology project of Jiangxi Health Commission (No. 202110028), Double thousand talents program of Jiangxi province (No. jxsq2019101021), and Young Talent Research and Cultivation Fund of the First Affiliated Hospital of Nanchang University (No. YFYPY202012).

## Conflict of interest

The authors declare that the research was conducted in the absence of any commercial or financial relationships that could be construed as a potential conflict of interest.

## Publisher's note

All claims expressed in this article are solely those of the authors and do not necessarily represent those of their affiliated organizations, or those of the publisher, the editors and the reviewers. Any product that may be evaluated in this article, or claim that may be made by its manufacturer, is not guaranteed or endorsed by the publisher.
